# Activation of RhoA/Rho-kinase pathway accounts for pulmonary endothelial dysfunction in patients with chronic obstructive pulmonary disease

**DOI:** 10.1002/phy2.105

**Published:** 2013-10-20

**Authors:** Yihua Bei, Sy Duong-Quy, Thong Hua-Huy, Pierre Dao, Nhat-Nam Le-Dong, Anh Tuan Dinh-Xuan

**Affiliations:** 1 Medical School, Assistance Publique Hôpitaux de Paris, Service de Physiologie, Paris Descartes UniversityEA 2511, Hôpital Cochin, 27 Rue du faubourg Saint-Jacques, 75014, Paris, France; 2 Clinical and Translational Research Center, Tongji University School of Medicine and Shanghai East Hospital150 Jimo Road, Shanghai, 200120, China

**Keywords:** COPD, endothelial dysfunction, eNOS, RhoA, Rho-kinases

## Abstract

Recent evidence suggests that activation of RhoA/Rho-kinase accounts for systemic and pulmonary endothelial dysfunction in smokers with normal lung function. However, its role in patients with chronic obstructive pulmonary disease (COPD) has not yet been investigated. The aim of this study was to evaluate the regulation of RhoA/Rho-kinase pathway and pulmonary endothelial dysfunction in patients with COPD. Pulmonary arteries were obtained from nonsmokers (control subjects) and patients with nonhypoxemic and hypoxemic COPD (*n* = 6–7/group). Endothelium-dependent and -independent relaxations were evaluated by acetylcholine and sodium nitroprusside, respectively. Gene and protein expressions of endothelial nitric oxide synthase (eNOS) were measured by RT-PCR, Western blot, and immunohistochemistry. Nitrate, cGMP, and endothelin-1 (ET-1) concentrations, as well as Rho-kinase activity were measured by ELISA. Protein expressions of total RhoA and GTP-RhoA were measured by Western blot and pull-down assay, respectively. Endothelium-dependent relaxation, and nitrate and cGMP levels were significantly reduced in pulmonary arteries of COPD patients as compared with control subjects. Conversely, activity of RhoA/Rho-kinase was increased in pulmonary arteries of COPD patients as compared with control subjects. In patients with COPD, pulmonary endothelial dysfunction was related to the downregulation of eNOS activity and upregulation of RhoA/Rho-kinase activity.

## Introduction

The prevalence of chronic obstructive pulmonary disease (COPD) has increased constantly since the last 20 years. In 2020, COPD will be the third leading cause of death worldwide after cardiac ischemia and vascular cerebral diseases (Murray and Lopez 1997; Lopez et al. 2006). In early stages of disease, COPD is characterized by airflow obstruction, mucus secretion, and airway inflammation. In advanced stages, COPD is marked by severe airflow limitation, lung parenchymal destruction, and pulmonary vascular remodeling. In these stages, pulmonary hypertension (PH) is often detected in COPD patients. It is suggested that chronic hypoxia-induced vasoconstriction and vascular hypertrophy are the principal causes of PH in patients with COPD (Barberà et al. 2003).

However, the structural changes in pulmonary arteries are not exclusive to patients with advanced COPD, as they have also been observed in patients with mild-to-moderate COPD and even in smokers with normal lung function (Barberà et al. 1994; Santos et al. 2002). These changes are principally characterized by the thickness of vascular walls and usually associated with impaired endothelial function resulting in altered modulation of vascular tone and permeability (Peinado et al. 1998). Increasing evidence demonstrated that the harmful effect of cigarette smoke is involved in the vascular structural damage and functional disturbances of endothelial cells. Endothelial dysfunction is thought to be the early manifestation of pulmonary vascular remodeling before it progresses to established PH. Although the relation between the severity of COPD and endothelial dysfunction has been demonstrated (Dinh-Xuan et al. 1991; Peinado et al. 1998), its link with reduced activity of endothelial nitric oxide synthase (eNOS) has not yet been firmly established.

Previous studies demonstrated that the small G-protein RhoA, with its downstream effector Rho-kinase (ROCK-I or ROCKβ and ROCK-II or ROCKα), has been involved in systemic endothelial dysfunction in smokers with normal lung function (Noma et al. 2005; Hidaka et al. 2010). This pathway plays important roles in diverse cell functions such as cell migration and proliferation, smooth muscle cell contraction, and gene expression (Duong-Quy et al. 2013). Recently, the role of RhoA/Rho-kinase pathway in pulmonary endothelial dysfunction in healthy smokers has also been demonstrated (Duong-Quy et al. 2011). It has been suggested that this pathway can negatively regulate eNOS expression and activity, as well as nitric oxide (NO) bioavailability (Takemoto et al. 2002; Rikitake and Liao 2005). Inhibition of RhoA/Rho-kinase increases the eNOS mRNA stability and expression (Laufs and Liao 1998; Rikitake et al. 2005). Besides the effect of selective Rho-kinase inhibitors in the treatment of PH in animal models, the beneficial pleiotropic effect of statins (HMG-CoA reductase inhibitors) in patients with COPD has been recently published (Frost et al. 2007; Søyseth et al. 2007).

Although the role of the RhoA/Rho-kinase pathway in pulmonary endothelial dysfunction has been demonstrated in healthy smokers (Duong-Quy et al. 2011), its role in patients with COPD has not been clarified. The aim of this study was to evaluate the regulation of RhoA/Rho-kinase pathway and the role of eNOS in pulmonary endothelial dysfunction in patients with COPD.

## Material and Methods

### Chemical reagents, antibodies, and kits

All chemical reagents were purchased from Sigma-Aldrich (Saint-Quentin Fallavier, France) unless otherwise noted. Antibodies included anti-eNOS antibody (BD Transduction Laboratories, Lexington, KY), anti-RhoA and secondary horseradish peroxidase (HRP)-conjugated antibodies (Santa Cruz, CA), and secondary biotinylated antibody (Vector Laboratories, Burlingame, CA). Rho activation kit for pull-down assay was purchased from Stressgen Bioreagent Corp. (Victoria, BC, Canada), and Rho-kinase assay kit from CycLex Co., Ltd (Tera-Sawaoka Ina, Nagano, Japan). Nitric oxide synthase assay kit was purchased from Cayman Chemical Europe (Massy, France), cGMP Enzyme-Immunoassay Biotrak (EIA) system from GE Healthcare Life Sciences (Little Chalfont, UK), and Endothelin-1 (ET-1) (1–21) Enzyme-Immunoassay kit from Biomedica (Vienna, Austria).

### Patients and pulmonary arterial samples

Patients who underwent lung resection for cancer were enrolled in this study. Three groups of patients were included: never smokers with normal lung function (control group), smokers with COPD and without hypoxemia (nonhypoxemic COPD group), and hypoxemic smokers with COPD (hypoxemic COPD group). The COPD patients were defined by the standard criteria (ratio of forced expiratory volume in 1 sec [FEV_1_] to forced vital capacity [FVC] <0.7) (Vestbo et al. 2013). Hypoxemia was defined by partial pressure of oxygen (Pao_2_) <80 mmHg. This study had been approved by the French Ethics Committee. Patients were informed about the study and had given informed consents.

Proximal pulmonary arteries were carefully dissected from apparently normal lung resection. Arterial rings of 3–4 mm external diameter and 4–6 mm of length were cut and immediately placed in Krebs–Henseleit solution at 4°C for vascular relaxation study within half an hour. Other pulmonary arterial samples were conserved at −80°C for protein and RNA extractions or fixed in formalin and embedded in paraffin for immunohistochemistry (IHC). Expression and/or activity data refer to the whole artery segments.

### Assessment of endothelium-dependent and -independent relaxations

The relaxations of pulmonary arterial rings were measured in organ bath chambers (Emka Technologies, Paris, France). Isometric tensions were measured and recorded by Mac Intosh Performa 630-Software. Briefly, pulmonary arterial rings were stabilized with Krebs–Henseleit solution warmed at 37°C and gassed with 95% O_2_ and 5% CO_2_ for at least 30 min at 1.5*g* resting tension. After being precontracted with l-phenylephrine, arterial rings were exposed to accumulating concentrations of acetylcholine (ACh, 10^−9^–10^−4^ mol/L) for endothelium-dependent relaxation, and sodium nitroprusside (SNP, 10^−9^–10^−4^ mol/L) for endothelium-independent relaxation maneuvers. For each subject, four pulmonary arterial rings were independently studied and only rings that were able to display submaximal precontraction in response to l-phenylephrine were kept for subsequent relaxation studies.

### Protein extraction for Western blot and enzyme-linked immunosorbent assay

Pulmonary arterial samples previously stocked at −80°C were defrosted in RIPA buffer (Cell Signaling, Boston, MA) containing protease and phosphatase inhibitors (Roche Applied Science, Mannheim, Germany). Pulmonary artery samples were homogenized at 24,000 rpm for 40 sec on ice. The homogenates were then centrifuged at 15,000 rpm for 20 min at 4°C. Protein concentration was determined by BCA Protein Assay Kit (Thermo Scientific, Rockford, IL) and pulmonary arterial supernatants were kept at −80°C for further protein analysis.

### Western blot for eNOS and RhoA protein expressions

Equal amounts of proteins from pulmonary arterial supernatants were loaded and migrated into sodiumdodecyl sulphate polyacrylamide gel electrophoresis (SDS-PAGE) gels by electrophoresis and then transferred to polyvinylidene difluoride (PVDF) membranes (Immobilon-P). Western blot was performed using appropriate primary antibodies and HRP-conjugated secondary antibodies before visualization via chemiluminescence (GE Healthcare, Buckinghamshire, UK). Blot density was determined by Image Software System (Genius 2, Syngene, Cambridge, UK). Protein expressions were normalized with β-actin.

### Measurement of GTP-RhoA protein expression by pull-down assay

RhoA activation was determined via measuring the active form of RhoA (GTP-RhoA) by immunoprecipitation. This assay used a GST fusion protein containing the Rho-binding domain (RBD) of mouse Rhotekin to affinity precipitate GTP-RhoA from lysates (500 μg of total proteins). After pull-down assay, 25 μL of eluted samples were separated by SDS-PAGE gels, then transferred to PVDF membranes and probed with antibody against RhoA. Blot density was evaluated as previously described. GTP-RhoA protein expression was normalized with β-actin.

### Measurement of nitrate, cGMP, endothelin-1, and myosin-binding subunit concentrations by ELISA

#### Nitrate concentration

NOS Assay Kit was used to measure newly synthesized NO from l-arginine by the action of eNOS in the presence of essential cofactors, according to the manufacturer's instructions. The final products of the reaction were nitrates, measured by colorimetric method (540 nm), which represented indirectly eNOS activity. Nitrate concentrations were determined via the standard curve.

#### cGMP concentration

cGMP concentration in pulmonary arteries, representing NO bioavailability, was measured by competitive EIA system according to the manufacturer's instructions. The assay combined the use of a peroxidase-labeled cGMP conjugate, a specific antiserum that can be immobilized on precoated microplates, and a stabilized substrate solution. It was based on competition between unlabeled cGMP and a fixed quantity of peroxidase-labeled cGMP, for a limited number of binding sites on a cGMP-specific antibody. cGMP concentrations were determined via a standard curve generated by provided cGMP protein standards.

#### ET-1 concentration

ET-1 concentration in pulmonary arteries was quantified by using ET-1 (1–21) kit. Briefly, 50 μL of supernatants of each sample and 200 μL of detection antibody reagent were deposed in duplicate in microtiter plate strips precoated with polyclonal antiendothelin antibody. After overnight incubation, the complexes were washed and then, incubated with 200 μL of conjugate and substrate reagents (HRP). Microtiter plate strips were read at 450-nm absorbance with correction at 620 nm. ET-1 concentrations were obtained from the standard curve.

#### MBS concentration

ROCK activity in pulmonary arterial supernatants (containing phosphatase inhibitors as previously detailed in protein extraction) was determined using CycLex Rho-kinase assay kit according to the manufacturer's protocol. Plates were precoated with a substrate corresponding to the recombinant C-terminus of MBS (myosin-binding subunit of myosin phosphatase), which contains a threonine residue that can be phosphorylated by ROCK or DMPK family members. ROCK in supernatants phosphorylated Thr-697 of the substrate, and the phosphorylated MBS was detected with specific HRP-conjugated antiphospho-MBS (Thr-697) antibody via spectrophotometric analysis (450 nm) of color changes in tetra-methylbenzidine (TMB).

### Immunohistochemistry for eNOS expression

For immunohistochemical staining of pulmonary arteries, paraffin sections were performed as follows. Endogenous peroxidase activity was reduced using 0.3% hydrogen peroxide in methanol for 30 min, then antigen unmask by sodium citrate solution (10 mmol/L, pH 6.0) was heated at 100°C for 20 min. Sections were then covered with 10% normal serum block in PBS/1% BSA (blocking buffer) for 2 h and incubated with primary anti-eNOS antibody (dilution 1:200, overnight, 4°C). Negative control sections were incubated in blocking buffer alone without primary antibody. Bound primary antibodies were detected using biotinylated antibody (dilution 1:200, 1 h, room temperature), coupled with preformed avidin–biotin (RTU Vectastain Kit, Vector Laboratories, Burlingame, CA) and DAB peroxidase substrate (3, 3′-diaminobenzidine). Sections were counterstained with hematoxylin. The eNOS expression was evaluated in endothelial cells by using intensity scores, assigned as no staining (0), focal staining (Barberà and Blanco 2009), diffuse weak staining (Barberà et al. 2001), diffuse moderate staining (Barberà et al. 2003), and diffuse strong staining (Barberà et al. 1994). At least 10 counted images were performed for each patient.

### RNA isolation, cDNA synthesis, and real-time polymerase chain reaction

Transcript levels of eNOS in pulmonary arteries were determined by quantitative real-time polymerase chain reaction (qRT-PCR) using LightCycler® 480 System (Roche Applied Sciences) and normalized to cyclophilin. Briefly, total RNA was extracted from frozen arterial tissues (50 mg/sample) using Trizol Reagent (Biomedica, Vienna, Austria) and quantified by a NanoDrop™ 1000 Spectrophotometer (Thermo Scientific). Reverse transcription reactions were performed on 1 μg of total RNA for each sample, using SuperScript® First-Strand Synthesis System (Invitrogen, Cergy-Pontoise, France). For qRT-PCR, all samples were processed in duplicate and results were accepted if variation coefficient was less than 0.2. Oligonucleotide primers were designed and purchased from Eurogentec (Liège, Belgium) as listed in Table 1. For qRT-PCR, samples were denatured at 95°C for 10 min, followed by 45 cycles of 25 sec (95°C for 10 sec, 60°C for 5 sec, and 72°C for 10 sec), fusion at 68°C for 30 sec, and cooling at 45°C for 10 sec. Relative quantification values of the target genes were standardized according to the value of cyclophilin as housekeeping gene.

**Table 1:** Oligonucleotide primers used in real-time PCR

Gene	Forward primers	Reverse primers
eNOS	TAA-GCA-GGC-CTG-GCG-CAA-CG	AGA-CCT-GCA-GTC-CCG-GGC-AT
Cyclophilin	ACC-GTG-TTC-TTC-GAC-ATT-GCC-GT	TGC-TGT-CTT-TGG-GAC-CTT-GTC-TGC

### Statistical analysis

Statistical comparisons were performed with t-Student test or Fisher's exact test between two groups or with analysis of variance (ANOVA) among more than three groups by using the SPSS 16.0 software (Chicago, IL). Data were presented as means ± SEM. Differences were considered significant with *P* < 0.05.

## Results

### Clinical and functional characteristics

Three groups of patients who met the inclusion criteria were constituted (6–7 subjects per group). Their clinical and functional characteristics are presented in Table 2. The patients with hypoxemic COPD had greater tobacco consumption and higher air flow obstruction (FEV_1_%) than those with nonhypoxemic COPD (*P* < 0.05). The ET-1 concentration in pulmonary arterial supernatants in hypoxemic COPD group was significantly higher than that in control group and nonhypoxemic COPD group (6.16 ± 0.83 vs. 1.43 ± 0.26 and 3.42 ± 0.63 fmol/mL; *P* < 0.001 and *P* < 0.05, respectively) (Table 2).

**Table 2:** Clinical and functional characteristics of patients

Variables	Controls (*n* = 7)	Nonhypoxemic COPD (*n* = 7)	Hypoxemic COPD (*n* = 6)	*P-*values
Age, years	59 ± 3.4	58 ± 4.2	60 ± 3.3	NS^1^,^2^,^3^
Male/female	4/3	5/2	4/2	–
Tobacco, packet-year	0	32 ± 2.3	41 ± 2.8	<0.05^3^
FEV_1_,% pred	91 ± 2.6	82 ± 3.8	68 ± 4.5	<0.05^1^; <0.01^2^; <0.05^3^
FEV_1_/FVC,%	84 ± 2.3	65 ± 1.5	61 ± 2.4	–
DLCOc,% pred	88 ± 3.5 (*n* = 4)	90 ± 4.5 (*n* = 4)	74 ± 5.8 (*n* = 3)	NS^1^; <0.05^2^,^3^
Pao_2_, mmHg	90 ± 1.9	84 ± 2.6	70 ± 2.0	NS^1^; <0.001^2^; <0.01^3^
Paco_2_, mmHg	38 ± 1.9	40 ± 1.9	42 ± 1.6	NS^1^,^2^,^3^
sPAP, mmHg	30 ± 1.8 (*n* = 5)	29 ± 2.2 (*n* = 5)	31 ± 1.5 (*n* = 4)	NS^1^,^2^,^3^
ET-1, fmol/mL	1.43 ± 0.26	3.42 ± 0.63	6.16 ± 0.83	<0.01^1^; <0.001^2^; <0.05^3^

% pred, percentage of predicted value; FEV_1_, forced expiratory volume in 1 sec; FVC, forced vital capacity; DLCOc, diffusing capacity of monoxide carbon corrected with hemoglobin; sPAP, systolic pulmonary arterial pressure; ET-1, endothelin-1; NS, no significant difference.

1 nonhypoxemic COPD versus controls

2 hypoxemic COPD versus controls

3 hypoxemic COPD versus nonhypoxemic COPD

### Endothelium-dependent and -independent relaxation

**Figure 1:**
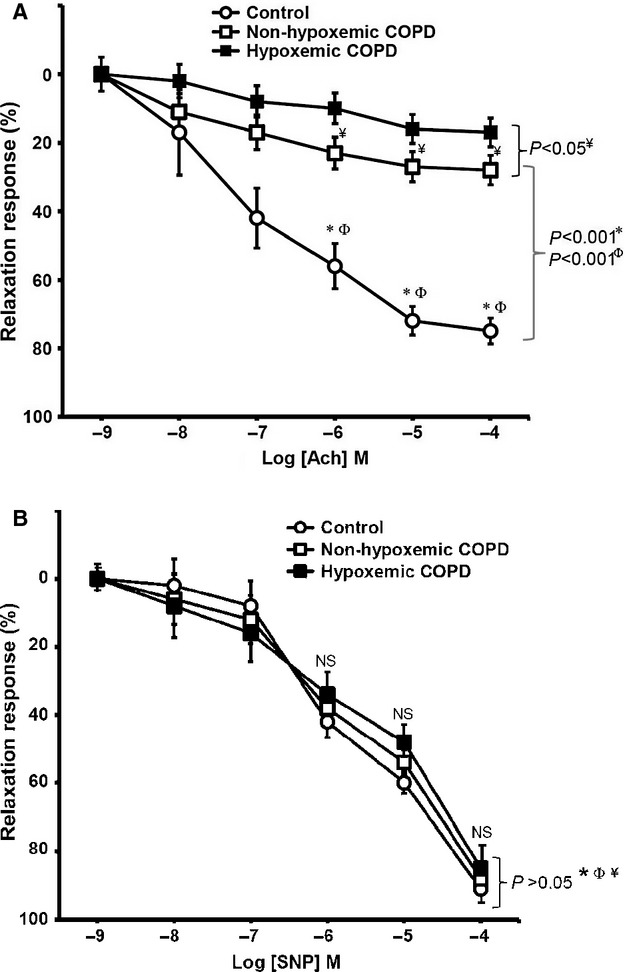
(A) Reduced endothelium-dependent relaxation in response to increasing concentrations of acetylcholine (ACh) in pulmonary arteries from COPD patients. *, control (*n* = 7) versus nonhypoxemic COPD (*n* = 7); Φ, control versus hypoxemic COPD (*n* = 6); ¥, nonhypoxemic COPD versus hypoxemic COPD. (B) Endothelium-independent relaxation in response to increasing concentrations of sodium nitroprusside (SNP). *, control (*n* = 7) versus nonhypoxemic COPD (*n* = 7); Φ, control versus hypoxemic COPD (*n* = 6); ¥, nonhypoxemic COPD versus hypoxemic COPD; NS, no significant difference.

The percentage of maximal relaxation in response to ACh (10^−6^–10^−4^ mol/L) for pulmonary endothelium-dependent relaxation was significantly lower in patients with hypoxemic COPD and nonhypoxemic COPD in comparison with control subjects (at 10^−5^ mol/L: 16 ± 4% and 27 ± 5% vs. 72 ± 9%; *P* < 0.001 and *P* < 0.001, respectively) (Fig. 1A). The difference in endothelium-dependent relaxation between nonhypoxemic COPD group and hypoxemic COPD group was significant (*P* < 0.05) (Fig. 1A).

Whatever the dose, pulmonary endothelium-independent relaxations in response to SNP were similar and not significantly different among three groups (*P* > 0.05) (Fig. 1B).

### eNOS immunostaining and eNOS gene and protein expressions

**Figure 2:**
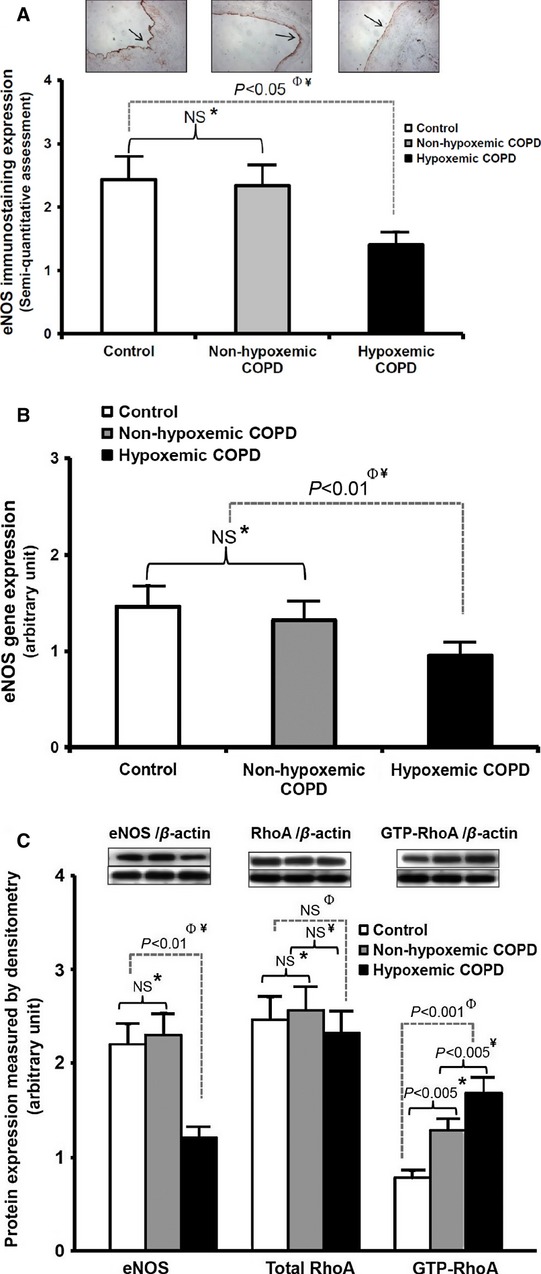
(A) Reduced immunostaining expression of eNOS in pulmonary arteries from hypoxemic COPD patients. Images on top: eNOS immunostaining of proximal arterial rings with magnification ×5. *, control (*n* = 7) versus nonhypoxemic COPD (*n* = 7); *Φ*, control versus hypoxemic COPD (*n* = 6); ¥, nonhypoxemic COPD versus hypoxemic COPD; NS, no significant difference. (B) Reduced gene expression of eNOS measured by RT-PCR in pulmonary arteries from hypoxemic COPD patients. *, control (*n* = 7) versus nonhypoxemic COPD (*n* = 7); *Φ,* control versus hypoxemic COPD (*n* = 6); ¥, nonhypoxemic COPD versus hypoxemic COPD; NS, no significant difference. (C) Protein expression of eNOS, total RhoA, and GTP-RhoA in pulmonary arteries measured by Western blot and pull-down assay. *, control (*n* = 7) versus nonhypoxemic COPD (*n* = 7); *Φ*, control versus hypoxemic COPD (*n* = 6); *¥*, nonhypoxemic COPD versus hypoxemic COPD; NS, no significant difference.

Semiquantitative assessment of positive staining to eNOS showed that eNOS expression in pulmonary arteries of patients with hypoxemic COPD was significantly lower than that of control subjects and patients with nonhypoxemic COPD (*P* < 0.05 and *P* < 0.05, respectively) (Fig. 2A). There was no significant difference in eNOS immunostaining between control group and nonhypoxemic COPD group (*P* > 0.05) (Fig. 2A).

Results of relative quantification showed that eNOS gene expression in pulmonary arteries of patients with hypoxemic COPD was significantly lower than that of control subjects and patients with nonhypoxemic COPD (*P* < 0.01 and *P* < 0.01, respectively) (Fig. 2B). There was no significant difference in eNOS gene expression between control group and nonhypoxemic COPD group (*P* > 0.05) (Fig. 2B).

Western blot analyses of eNOS protein expression in pulmonary arteries showed that eNOS expression in hypoxemic COPD group was significantly lower than that in control group and nonhypoxemic COPD group (*P* < 0.01 and *P* < 0.01, respectively) (Fig. 2C). There was no significant difference in eNOS protein expression between control group and nonhypoxemic COPD group (*P* > 0.05) (Fig. 2C).

### RhoA and GTP-RhoA protein expressions

Total RhoA protein expression in pulmonary arterial supernatants was measured by Western blot. There was no significant difference in total RhoA protein expression among three groups (*P* > 0.05) (Fig. 2C).

The activity of RhoA in pulmonary arterial supernatants was evaluated via measuring the active form of RhoA (GTP-RhoA) by immunoprecipitation. Results of pull-down assay showed that GTP-RhoA protein expression in nonhypoxemic and hypoxemic COPD groups was significantly higher than that in control group (*P* < 0.005 and *P* < 0.001, respectively) (Fig. 2C). Between COPD groups, GTP-RhoA protein expression in hypoxemic COPD group was still higher than that in nonhypoxemic COPD group (*P* < 0.005) (Fig. 2C). The percentage of GTP-RhoA/total RhoA protein expression in nonhypoxemic and hypoxemic COPD groups was significantly higher than that in control group (38 ± 12% and 56 ± 10% vs. 18 ± 7%; *P* < 0.01 and *P* < 0.001, respectively).

### Nitrate, cGMP, and MBS concentrations

**Figure 3:**
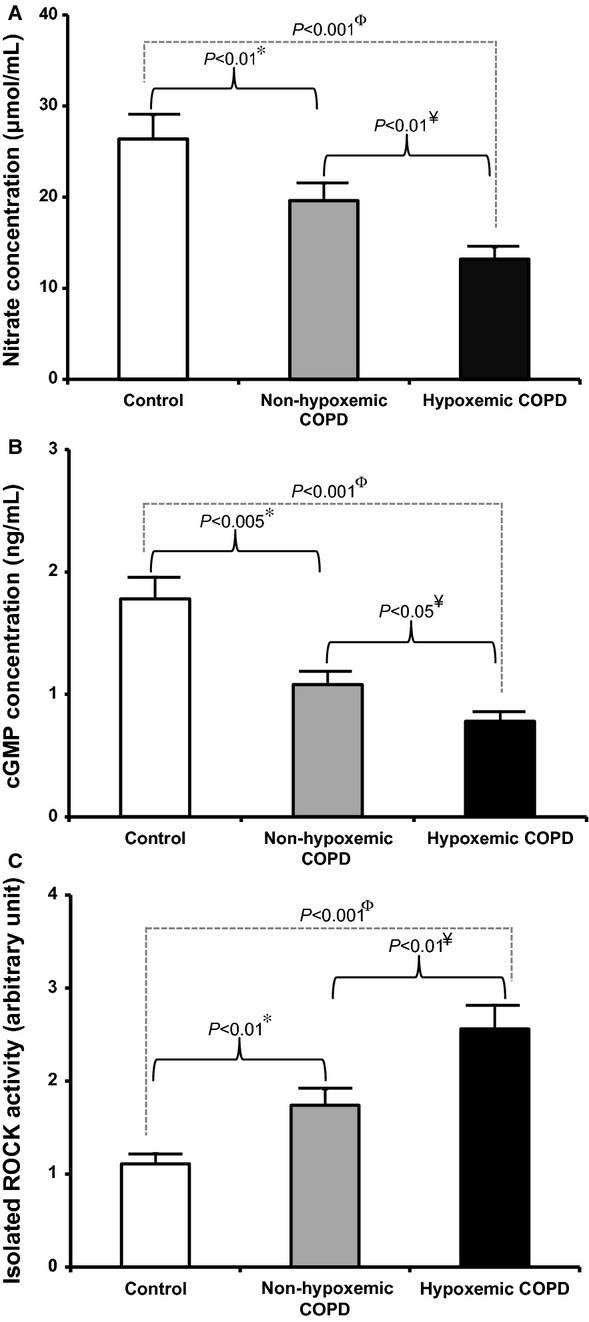
(A) Reduced concentration of nitrate measured by enzyme-linked immunosorbent assay (ELISA) in pulmonary arterial supernatants from COPD patients. *, control (*n* = 7) versus nonhypoxemic COPD (n = 7); Φ, control versus hypoxemic COPD (*n* = 6); *¥*, nonhypoxemic COPD versus hypoxemic COPD. (B) Reduced concentration of cGMP measured by ELISA in pulmonary arterial supernatants from COPD patients. *,: control (*n* = 7) versus nonhypoxemic COPD (*n* = 7); Φ, control versus hypoxemic COPD (*n* = 6); *¥*, nonhypoxemic COPD versus hypoxemic COPD. (C) Increased isolated ROCK activity measured by ELISA in pulmonary arterial supernatants from COPD patients. *, control (*n* = 7) versus nonhypoxemic COPD (*n* = 7); Φ, control versus hypoxemic COPD (*n* = 6); ¥, nonhypoxemic COPD versus hypoxemic COPD.

Nitrate concentration in pulmonary arterial supernatants in nonhypoxemic and hypoxemic COPD groups was significantly lower than that in control group (19.68 ± 2.34 and 13.20 ± 2.30 vs. 26.48 ± 3.66 μmol/mL; *P* < 0.01 and *P* < 0.001, respectively) (Fig. 3A). The difference in nitrate concentration between nonhypoxemic and hypoxemic COPD groups was still significant (*P* < 0.01).

Concentration of cGMP in pulmonary arterial supernatants in nonhypoxemic and hypoxemic COPD groups was significantly lower than that in control group (1.06 ± 0.11 and 0.78 ± 0.10 vs. 1.77 ± 0.15 ng/mL; *P* < 0.005 and *P* < 0.001, respectively) (Fig. 3B). The difference in cGMP concentration between nonhypoxemic and hypoxemic COPD groups was still significant (*P* < 0.05).

The activity of Rho-kinase was evaluated by measuring the phosphorylated form of MBS. The relative amounts of phosphorylated MBS in pulmonary arterial supernatants in nonhypoxemic and hypoxemic COPD groups were significantly higher than those in control group (*P* < 0.01 and *P* < 0.001, respectively) (Fig. 3C). The difference in MBS concentration between nonhypoxemic and hypoxemic COPD groups was still significant (*P* < 0.01).

## Discussion

Although the upregulation of RhoA/Rho-kinase pathway in smokers with normal lung function has been demonstrated, the role of this pathway in patients with COPD has not been well studied. The results of this study showed that: (1) patients with COPD had an impairment of endothelium-dependent relaxation that most probably resulted from reduced eNOS activity; (2) RhoA/Rho-kinase activity in pulmonary arteries was increased in patients with COPD, especially in those with high tobacco consumption, hypoxemic status, and high ET-1 concentration; (3) increase in RhoA activity (GTP-RhoA) in patients with COPD was accompanied by impairment of eNOS expression and activity; (4) all these impairments were more severe in hypoxemic COPD compared with nonhypoxemic COPD; and (5) eNOS gene and protein expressions were only decreased in patients with hypoxemic COPD.

In systemic circulation, endothelial cells play a crucial role in the regulation of vascular tone. The endothelial dysfunction is principally related to the abnormality in synthesis and release of vasoactive mediators such as NO or ET-1, leading to the alteration of vasodilatation and vasoconstriction balance (Budhiraja et al. 2004). The endothelial dysfunction associated with cigarette smoke is considered as the initial event in the pathogenesis of PH in COPD, which even precedes the vascular structural changes and PH establishment (Barberà and Blanco 2009). The results of this study showed that endothelium-dependent relaxation was reduced in patients with COPD, whereas endothelium-independent relaxation was normal as compared with control subjects. Especially, the impairment of endothelium-dependent relaxation was even more severe in hypoxemic COPD in comparison to nonhypoxemic COPD (Fig. 1A). Under the effect of cigarette smoke and chronic hypoxia, endothelial dysfunction can be present not only in severe COPD (Dinh-Xuan et al. 1991) but also in mild-to-moderate COPD (Peinado et al. 1998, 1999) and even in healthy smokers (Barberà et al. 2001; Duong-Quy et al. 2011).

In this study, the impairment of endothelium-dependent relaxation of proximal pulmonary arteries was present in patients with COPD regardless of existence of hypoxemia; however, the reduction in eNOS protein and gene expressions was only significant in patients with hypoxemic COPD (Fig. 2). Our present results are slightly different from those of previous studies, in which the decrease in eNOS expression has been demonstrated in patients with mild-to-moderate COPD and even in smokers with normal lung function (Barberà et al. 2001). These controversial results might be due to the different structures of pulmonary arteries studied: entire lung tissue or parenchymal pulmonary arteries (external diameter of 1.5–2 mm) in previous studies versus proximal pulmonary arteries (external diameter of 3–4 mm) in the present study. Our previous published study measuring proximal pulmonary arteries showed that endothelium-dependent relaxation is impaired in healthy smokers when compared with nonsmokers, whereas eNOS protein expression is not significantly different between smokers and nonsmokers (Duong-Quy et al. 2011). Thus, the results concerning the regulation of eNOS expression in healthy smokers or patients with mild-to-moderate COPD remain controversial, whereas the reduction in eNOS expression in pulmonary arteries in severe COPD or in COPD with PH has already been confirmed (Peinado et al. 2008). However, in our present study, the mean pulmonary arterial pressure (mPAP) measured by transthoracic echography in small sample size of patients was not significantly different among three groups (Table 2).

In this study, eNOS activity was evaluated by measuring the production of nitrate in pulmonary arterial supernatants, which was significantly reduced in patients with nonhypoxemic and hypoxemic COPD as compared with control subjects (Fig. 3A). We then measured cGMP concentration, which represents NO bioavailability. The results showed that cGMP concentration in pulmonary arterial supernatants was also significantly reduced in patients with nonhypoxemic and hypoxemic COPD as compared with control subjects (Fig. 3B), suggesting that the bioactivity of NO on soluble guanylate cyclase (sGC) in the formation of cGMP was impaired. Furthermore, the impairment of eNOS activity (nitrate concentration) as well as NO bioavailability (cGMP concentration) in pulmonary arteries was more severe in hypoxemic COPD than nonhypoxemic COPD (Fig. 3A and B). We hypothesize that endothelial dysfunction of proximal pulmonary arteries in patients with mild-to-moderate COPD in this study might be principally related to reduced eNOS activity rather than eNOS expression.

Although the molecular mechanism by which eNOS expression and activity were negatively regulated in pulmonary arteries of COPD patients had not been studied in this study, it might be due to the harmful effect of cigarette smoke on pulmonary endothelial cells via inflammatory mediators and free radicals as demonstrated previously (Raij et al. 2001; van der Vaart et al. 2004). Su et al. (1998) showed that cigarette smoke extract (CSE) produces an irreversible inhibition of eNOS expression and activity in pulmonary arterial endothelial cells. More recently, Zhang et al. (2006) showed that CSE-induced reduction in eNOS activity and NO production/bioavailability is associated with the production of reactive oxygen species (ROS) as well as an altered arginine metabolism in pulmonary arterial endothelial cells (Zhang et al. 2006). However, in this study, among COPD patients (13 subjects), there were three former smokers who had a hypoxemia and moderate COPD with an impairment of eNOS activity (low concentration of nitrate and cGMP), which suggests that besides harmful effect of cigarette smoke, there are also other intrinsic factors which may be involved in the impairment of eNOS expression and activity in patients with COPD.

The role of RhoA/Rho-kinase pathway in systemic endothelial dysfunction in smokers with normal lung function has already been demonstrated (Noma et al. 2005; Hidaka et al. 2010). Besides, the increase in RhoA/Rho-kinase activity in pulmonary arteries from smokers with normal lung function has also been confirmed by our previously published study (Duong-Quy et al. 2011). The activation of RhoA/Rho-kinase pathway plays important roles in regulating many cell functions, such as proliferation, migration, apoptosis, contraction, as well as gene expression and endothelial dysfunction (Duong-Quy et al. 2013), which can be involved in the pathogenesis of COPD (Storck and Wojciak-Stothard 2013). In this study, we further investigated the regulation of RhoA/Rho-kinase pathway in patients with COPD. We first evaluated the activity of RhoA in pulmonary arteries by measuring the active form of RhoA (GTP-RhoA). The results showed that while total RhoA protein expression was similar among three groups, GTP-RhoA protein expression was significantly increased in patients with COPD (Fig. 2C). The increase in RhoA activity was accompanied by an increase in Rho-kinase activity, confirmed by measuring the phosphorylated form of MBS (Fig. 3C). Moreover, the activity of RhoA/Rho-kinase pathway in pulmonary arteries was even higher in patients with hypoxemic COPD than those with nonhypoxemic COPD, which suggests that hypoxia might play a potential role in the upregulation of RhoA/Rho-kinase pathway in COPD. The increase in RhoA/Rho-kinase activity associated with chronic hypoxia has already been demonstrated in animal models of PH (Nagaoka et al. 2004; Hyvelin et al. 2005). Although the mechanism by which RhoA/Rho-kinase pathway is activated in chronic hypoxia has not been well understood, previous studies indicate that ROS are importantly involved in the activation of multiple intracellular signaling cascades including RhoA/Rho-kinase pathway (Jin et al. 2004). Recently, the role of ROS in the upregulation of RhoA/Rho-kinase pathway in chronic hypoxia-induced PH in rats has been demonstrated (Jernigan et al. 2008). However, the mechanism by which the RhoA/Rho-kinase pathway is activated in COPD via these factors needs to be further studied.

In this study, upregulation of RhoA/Rho-kinase activity was accompanied by downregulation of eNOS expression and activity in patients with COPD. These results reveal potential interaction between RhoA/Rho-kinase pathway and eNOS/NO/cGMP pathway in the pathogenesis of COPD. Previous studies showed that RhoA/Rho-kinase activation can negatively regulate eNOS expression and activity, as well as NO bioavailability, whereas RhoA/Rho-kinase inhibition can increase eNOS mRNA stability (Laufs and Liao 1998; Laufs et al. 1999; Takemoto et al. 2002; Rikitake and Liao 2005). Besides, Rho-kinase inhibitors can also enhance the phosphorylation and activation of transcriptional factors, leading to the increase in NO production (Wolfrum et al. 2004). However, the mechanism by which RhoA/Rho-kinase pathway interferes with eNOS expression and activity in pulmonary arteries from patients with COPD should be further clarified.

## Conclusions

This study showed the presence of endothelial dysfunction in pulmonary arteries of patients with COPD where downregulation of eNOS activity and upregulation of RhoA/Rho-kinase activity also occurred. The interaction between RhoA/Rho-kinase pathway and eNOS/NO/cGMP pathway in endothelial dysfunction in the pathogenesis of COPD needs to be further studied.
